# Successful Repair of TEF and DORV in a Child in a Resource-Limited Setting

**DOI:** 10.1155/2023/1095670

**Published:** 2023-02-14

**Authors:** Eru Sujakhu, Rajendra Shilpakar, Dhruba Shrestha

**Affiliations:** Siddhi Memorial Hospital (For Women and Children), Bhaktapur, Nepal

## Abstract

VACTERL association is typically defined by the presence of at least three of the congenital malformations that make up the term including: vertebral defects, anal atresia, cardiac defects, tracheoesophageal fistula (TEF), renal anomalies, and limb deformities. Patients with VACTERL are typically managed through immediate-postnatal-surgical correction of the specific congenital anomalies (typically anal atresia, specific types of cardiac malformations, and/or TEF), followed by long-term medical management of the congenital malformations. Although congenital anomalies might have long-lasting effects, the prognosis can be positive when the best surgical remedy is possible. Here, we present a case of 5 years female that is a known case of VACTERL Status Post (S/P)TEF repair, S/P double outlet right ventricle repair at sixth day and fifth month of life. This child managed to survive despite being operated in a resource-limited setting.

## 1. Introduction

The term “VACTERL association” refers to a collection of multisystem congenital defects including vertebral deformities (V), anorectal malformations (A), cardiac malformations (C), tracheoesophageal fistulas (TEF) with or without esophageal atresia (EA), renal abnormalities (R), and limb abnormalities (L) [1]. It affects between 1 in 10,000 and 1 in 40,000 live-born newborns [2]. Anomalies of the esophagus and trachea account for 70% of all cases, whereas 68.9% and 65.6% of patients have malformations of the vertebrae/ribs and cardiovascular system, respectively [3]. Among all the types of TEF, EA with TEF coexists in 90% of cases and is usually diagnosed and surgically treated in early infancy [4]. TEF patients frequently experience feeding issues, respiratory distress, cyanosis, coughing, and drooling. [5]. Because cardiac anomalies are associated with EA–TEF with a reported incidence of 13–40%, all EA–TEF patients should undergo echocardiography (ECHO) [6]. Better perioperative care is enhancing the survival of those with congenital heart disease (CHD), it still represents a substantial mortality risk factor of 40–50% (Spitz classification, Gr II) [7].

Double outlet right ventricle (DORV) is a type of ventriculoarterial connection in which both major vessels either totally or primarily emerge from the right ventricle [8]. Similar to other complicated CHDs, DORV may manifest as a single heart defect, in conjunction with additional cardiac defects, or in conjunction with extra-cardiac malformations [9]. It occurs in approximately 3–9/100,000 live births [8]. The relationship between the great arteries and the interventricular connection, the presence of an obstruction in the outflow tract, and the relative systemic-to-pulmonary artery resistance, which together influence the hemodynamic condition, determine the clinical symptoms of a DORV. [10]. Majority of individuals with DORV present either during the prenatal or perinatal period of life [7]. Patients with these associated abnormalities typically require surgical intervention of TEF prior to the correction or palliation of the cardiac defect [10].

## 2. Case Presentation

**Figure 1:**
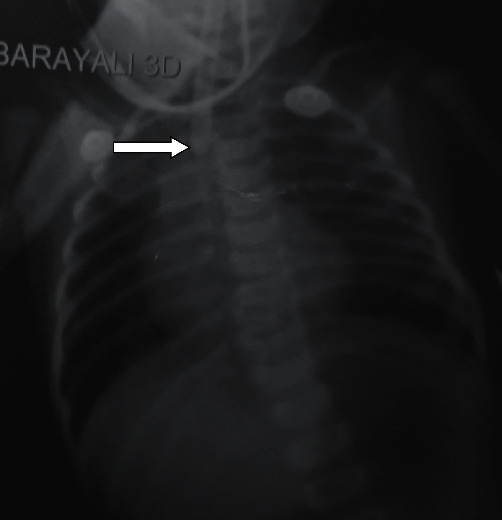
X-ray chest prior to the TEF repair showed the red rubber catheter (white arrow) in the proximal esophagus with presence of gas in the stomach and intestine consistent with TEF.

Our case was born to a 34 years old G3P2 mother via emergency cesarean section at 39 weeks of gestation for fetal distress with moderate meconium stained fluid. The mother had a good antenatal history with regular intake of folic acid, iron, and calcium supplements with no other known comorbidities during and prior to her pregnancy. No previous pregnancies with congenital malformations were recorded, and family history was also unremarkable. Her antenatal scans were all unremarkable except for the evidence of polyhydramnios during the eighth month of her pregnancy. The baby was born with a weight of 2.5 kg and an Apgar score of 7/10 and 8/10 at 1 and 5 minutes, respectively, and 3 hours after delivery she started regurgitating the milk she was being fed. She was obviously under distress with evidence of tachypnea, nasal flaring, grunting, subcostal retraction, and frothiness from mouth. She was diagnosed to have TEF type C after doing an X-ray with red rubber catheter in situ (Figure 1). On further screening, she was found to have CHD (DORV, patent ductus arteriosus (PDA), and subaortic ventricular septal defect) and vertebral anomaly with scoliosis (Spitz Classification, Gr II).

With the consideration of VACTERL association, she was referred to the higher center for surgical repair of her TEF. Routine preoperative work-up included unremarkable laboratory values that included: white blood cells: 17000/mm^3^; neutrophils: 80%; lymphocytes: 16%; Hb: 17.8 gm/dl; platelets: 141,000/mm^3^; urea: 20 mg/dl; creatinine: 0.95 mg/dl; and calcium: 2.2 mg/dl. The patient was scheduled for repair of the fistula to precede her cardiac repair and, hence, underwent TEF repair on her sixth day of life. The fistula was divided and closed in a single setting.

**Figure 2:**
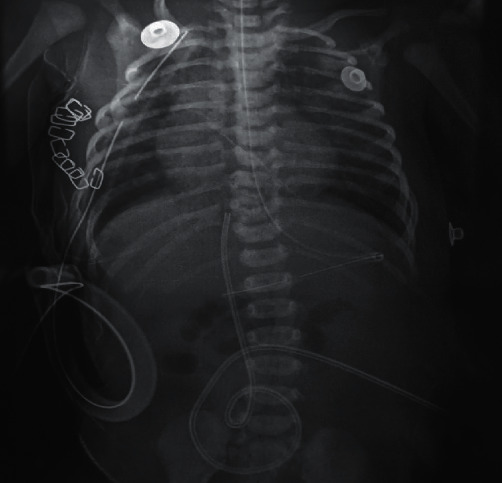
X-ray chest showed the nasogastric tube in the stomach after the successful repair of the TEF.

She was subsequently admitted to the neonatal intensive care unit for 21 days during which she had non-resolving pneumonia. She was kept under mechanical ventilation for five days with 40% FiO_2_, taking care to avoid over suctioning the endotracheal tube and positive pressure to prevent failure of the repair. Enteral feeding was established 7 days post-surgery after doing the methylene blue test to ensure that there was no anastomotic leak (Figure 2). The postoperative course was without complication, and she was discharged on 22nd day on digoxin, furosemide/spironolactone, and supplement medicines like iron, folic acid, calcium, and vitamins to continue.

She was first assessed at our center in her second month of life when she was admitted with the diagnosis of VACTERL, Status Post (S/P) TEF repair, DORV with mal-aligned ventricular septal defect (VSD), PDA with Congestive cardiac failure, and bilateral pneumonia with seizure. She presented with cough for 2 days and inconsolable cry for 1 day and on examination, she was ill-looking, weighed 2.66 kg, and had subcostal retraction, bilateral crepitation with SpO_2_ of 80%. Patient developed multiple episodes of generalized seizure during the admission. Electroencephalogram (EEG) showed frequent bilaterally independent parieto-occipito temporal epileptiform abnormalities with probable bilateral secondary synchrony. Due to these predisposing factors, the child was considered to have failure to thrive and had delayed milestones. After being treated with cefotaxime, gentamycin, and meropenem along with the continuation of her regular medications, she was discharged after 3 weeks with additional medicines phenobarbitone and enalapril. A repeat EEG was normal; hence, patient was weaned off the antiepileptic medications after a month of its initiation. She has been since then under regular follow up here with history of recurrent pneumonia that required multiple admissions.

**Figure 3:**
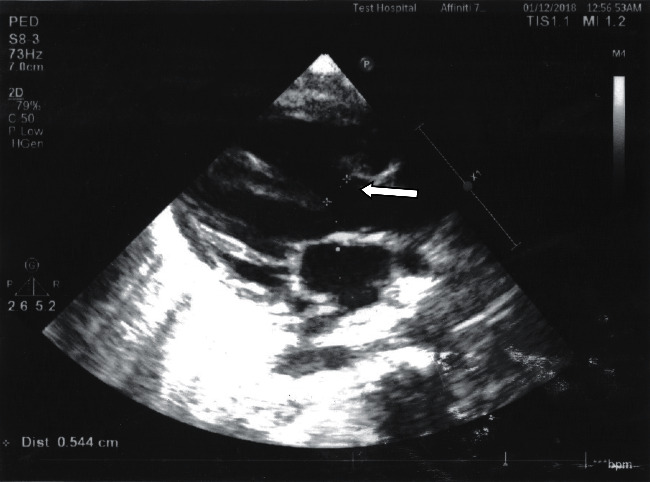
ECHO image showing large VSD with more than 50% overriding of aorta by interventricular septum (DORV; small white arrow).

**Figure 4:**
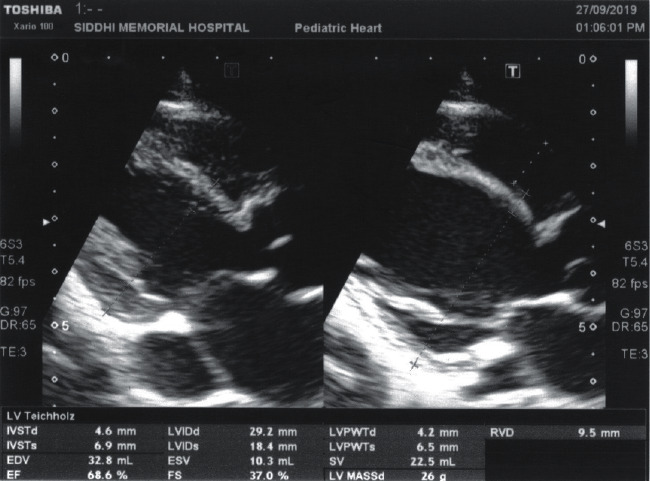
ECHO image after repair of VSD using PTFE patch.

Due to her non-resolving symptoms of pneumonia, she was referred to the cardiac center of our country at fifth month of her life, for surgical correction of her DORV (Figure 3). The procedure included ligation of PDA, right atriotomy, polytetrafluoroethylene mesh (PTFE) patch closure of VSD, repair of the tricuspid valve, and surgical closure of the patent foramen ovale after the establishment of cardiopulmonary bypass and induction of cardioplegic cardiac arrest. The postoperative course was uneventful. She was discharged 22 days after her hospital stay with furosemide and spironolactone. The medications were gradually tapered off with the continuation of furosemide for maintenance. The repeat ECHO findings were all normal (Figure 4).

As of this writing, the patient is doing fine with just mild psychomotor impairment, no long-term consequences, and recurring pneumonia. The routine follow-up examinations among blood and urine testing, chest and spinal X-rays, ECHO, and renal function are normal. The child is now 5 years old and is currently under budesonide and salbutamol Metered Dose Inhaler (MDI). Her vaccinations are all up-to-date. The patient experienced failure to thrive as an infant and has a mild psychomotor delay. Her growth trajectory had always remained below the third centile.

This is a rare, but successfully managed case with high mortality if not treated on time. Our country is an under-developed nation that often lacks the resources necessary to manage these cases. Despite being managed in the resource-limited setting, this child could survive and live a relatively normal life without significant residual cardiac disease.

## 3. Discussion

Our patient had subaortic VSD (5 mm) with a bidirectional shunt, an aortic override of 50%, and an ostium secundum atrial septal defect (4 mm) with a left to right shunt. In order to adequately oxygenate and ventilate the patient while waiting for her CHD to be treated, the TEF needed to be repaired immediately. Early surgery is preferred as gastric distension can lead to hemodynamic compromise, aspiration, hypoventilation, and aspiration prior to ligation of the TEF. Each of the fundamental component features must be tested for in patients who are considering VACTERL association, with the initial testing for each of the following features being the bare minimum: a complete medical history and physical examination by a competent physician, spinal Magnetic Resonance Imaging (MRI) or ultrasound, renal ultrasound, and blood and urine testing for renal function [6]. Every three to six months, if possible, the infant should be observed by a pediatrician who will also supervise the child's rehabilitation and prescribe any necessary adapted equipment [11]. DORV is a condition that cannot resolve itself. Therefore, palliative surgeries are only carried out on patients who need immediate care [12]. Preoperative evaluation of these patients must involve cardiology consultation and ECHO to identify the anatomic lesion, physiology, and coexisting abnormalities. Early diagnosis and aggressive treatment of associated anomalies, particularly cardiac malformations, resulted in significant decreases in mortality rates [13]. The care of patients is challenging since patients and families with VACTERL association symptoms are given scant information about long-term prognoses and outcomes. Like in our case, the parents were only directed towards the negative possible outcomes neglecting the fact that the child can also have a better life afterwards with continuity of medical treatment. According to each component feature of VACTERL association, treatment involves surgical correction of the congenital abnormality and the long-term management [6]. The staged, palliative bidirectional Glenn procedure has been used for patients with univentricular hearts or complex CHD, including DORV [15]. Early diagnosis, major advancements in pulmonary and enteral care, and improvements in nutritional support all have improved survival rates [7]. Though there was a delay in the surgical correction of the cardiac malformation in our case, the outcomes have been satisfactory and the patient is doing well as of this date. Majority of the patients with VACTERL association are born small and have difficulty with gaining weight. Patients, however, do tend to have normal development and normal intelligence [14].

## Data Availability

Data supporting this research article are available from the corresponding author or first author on reasonable request.

## Abbreviations

DORV: Double outlet right ventricle
VACTERL: Vertebral anomaly, anorectal malformation, cardiac anomaly, tracheo esophageal fistula, renal anomaly, and limb abnormality
VSD: Ventricular septal defect
ECHO: Echocardiography
PDA: Patent ductus arteriosus
EEG: Electroencephalogram
PTFE: Polytetrafluoroethylene mesh.
